# Physicochemical and sensory qualities of complemenatry meal made from sprouted and unsprouted sorghum, Irish potato and groundnut

**DOI:** 10.1002/fsn3.556

**Published:** 2017-12-19

**Authors:** Omolara R. Adegbanke, Toluwase A. Dada, Stephen A. Akinola, Temitope Akintuyi

**Affiliations:** ^1^ Department of Food Science and Technology Federal University of Technology Akure Ondo State Nigeria; ^2^ Department of Animal Health Food Safety & Toxicology Unit North‐West University Mafikeng Campus Mmabatho South Africa; ^3^ Microbiology Division Department of Biological Sciences North West University Mafikeng Campus Mmabatho South Africa

**Keywords:** Malnutrition, Nutritive value, Sprouted and Unsprouted, Weaning food

## Abstract

Weaning food was produced from the blends of sprouted and unsprouted sorghum–Irish potato, and groundnut flour. In the developed weaning foods, moisture content ranged from 8.44% to 12.70%, crude protein (7.40%–14.53%) crude ash (1.53%–1.77%), crude fiber (6.65%–6.88%), crude fat (3.31%–3.73%) and carbohydrate content (65.10%–69.15%). Sprouting and protein supplementation with groundnut improved the protein content of the formulated meals with values comparable to commercial sample (cereals). Mineral content reduced with sprouting, whereas the addition of Irish potato and groundnut increased the mineral content. Calcium ranged from 91.00% to 121.33% and potassium (487.33%–956.67%). Sample NSIG2 had the highest potassium. Tannin ranged from 0.11 to 0.64 mg/100 g; phytate (4.98–7.42 mg/100 g); and oxalate (0.36–0.98 mg/100 g). Peak viscosity ranged from 43.08 to 23.57 RVU, trough (41.08–22.50 RVU), breakdown viscosity (61–14), final viscosity (84.33–52.53 RVU), setback viscosity (41.33–89.00 RVU), and peak time (5.07–7.00) in both the sprouted and unsprouted sorghum–irish potato–groundnut flour, respectively. The pasting temperature of the weaning food blends ranged between 87.25 and 89.60°C with SIG0 and NSIG2 having the lowest and highest values, respectively. The study showed that complementary food products formulated from this locally available food commodities is a promising food and has good nutritive value.

## 1 INTRODUCTION

A child grows and develops physically, emotionally, cognitively, and socially when he is well‐nourished. Research has shown that adequate diet during infancy and early childhood is essential to the growth, health, and development of children to attain their full potential. Exclusive breastfeeding from a well‐nourished mother is adequate to meet the nutritional requirement of the infant in the first 6 months (WHO, 2004). After, it is expected that the breast milk is supplemented by complementary foods to meet their growth requirement. This is essential because, breast milk alone cannot provide the child with all the required nutrients after 6 months of age; in particular, iron, hence, the need to introduce complementary foods. Any food, liquid, semiliquid or solid given to an infant or child that complement breast milk is referred to or can be regarded as a complementary food (Kleinman, 2004). It is generally introduced between the ages of 6 months to 3 years old. Well‐breastfed infants are often able to maintain adequate growth through their sixth month, additional nutrients are required to complement or, in some cases, replace breastfeeding completely. The main concern is making sure that there is no gap between nutrient requirements and what a child is able to consume, absorb, and utilize.

Sorghum (*Sorghum bicolor* L Monech) is one of the leading cereal crops that are widely used as human food, poultry, cattle, and horse feed, as well as major source of energy, protein, vitamins, and minerals (Bolarinwa et al., 2015). Sorghum is remarkably significant to food security in Africa due to its distinctive drought‐resistant characteristics among other cereals and its ability to withstand periods of high temperature (FAO & ICRISAT, 1996). Sorghum products include expanded snacks, cookies, and ethnic foods.

Potato is a tuberous dicotyledonous crop grown all over the world because of its special role in human diet (Ikanone & Oyekan, 2014). Irish potatoes are mostly cross‐pollinated by insects such as bumblebees, and they are rich source of protein, carbohydrates, minerals, and vitamins (Hamilton et al., 2004).

Groundnut (*Arachis hypogea L*.) is a major annual oil seed crop and a good source of protein (Asibuo et al., 2008) in the tropics. The chemical composition of groundnut seed has been assessed in relation to protein level, fatty acid composition of cultivars to be a good source of protein and oil (Asibuo et al., 2008 & Eshun et al., 2013). Groundnut contains about 44%–55% oil, protein 22%–30%, riboflavin, minerals (Ca, Mg, K), and vitamins (Savage & Keenan, 1994).

The major problems associated with the infant during the transitional phase of weaning is the protein energy malnutrition (PEM), which is associated with wasting condition resulting from an inadequate protein or/and energy diet (Modu et al., 2013). Sorghum has a poor nutritional value due to its deficiency in Lysine, threonine, and tryptophan which can retard growth, development, decrease immunity, and weaken the hearts and lungs. This is due to the presence of antinutritional factors such as tannin and phytate which reduces nutrient utilization of plant products used for human foods (Habtamu & Negussie, 2014). Hence, the need to reduce this antinutrient by sprouting and develop a complementary meal that is rich in protein and essential micronutrients using locally available crops like groundnut and irish potato.

Therefore, the aim of this study was to formulate a weaning food from locally available materials (sorghum grains, Irish potato tubers, and groundnut) and evaluate its physicochemical and acceptability of the diet for infants.

## 2 MATERIALS AND METHODS

### 2.1 Sample collection and preparation

Sorghum grains, Irish potato, fresh groundnut grains were purchased from Erekesan market, Akure, Ondo State, Nigeria, whereas all other reagents were sourced at Food Science and Technology Laboratory, Federal University of Technology, Akure.

Weaning food blends was formulated from sorghum flour according to the method described by Bolarinwa et al. (2015). Sorghum grains were sorted to remove stones, dirt, and other extraneous materials. The cleaned grains was thoroughly washed and steeped in water for 12 h. The hydrated grains were spread on a moist jute bag which was sterilized by boiling for 30 min and the grains were allowed to germinate for 96 h. The germinated seeds were dried at 60°C in a cabinet dryer for 6 h to a moisture content of 10%–12% (Bolarinwa et al., 2015). The withered rootless grains were gently brushed off and the sprouted grains were dried milled, sieved through a 0–1 mm screen to obtain the sorghum flour and packaged in an air‐tight container.

Unsprouted sorghum flour was produced by sorting wholesome sorghum grains to remove dirt, stones, and other extraneous materials. It was weighed, washed thoroughly, drained and dried using cabinet dryer at 60°C for 6 h. It was then cooled and ground using hammer mill to pass through a 0–1 mm screen to obtain the sorghum flour.

Preparation of Irish potato flour was done by manually sorting potato tubers to remove bad ones from the lot. The sorted tubers were weighed, peeled, and sliced to facilitate the rate of drying and ease of milling operations. The sliced tubers were then immersed in 0.1% citric acid solution for 5 min to inactivate enzymes that may initiate a browning reaction. The blanched sliced tubers were drained and dried, milled, sieved into fine flour and packaged in an air‐tight container (Adeleke & Adedeji, 2012).

Preparation of Groundnut flour was done manually by first removing moldy, shriveled nuts and stones from groundnut grains, while the dust was removed by winnowing in a basket. The grains were dried using cabinet dryer at 60°C for 5 h to facilitate dehulling. The hulls were removed by rubbing in between palms. The groundnut was milled with the attrition milling machine and defatting was done in a Soxhlet extractor. The defatted cake was dried, milled, and sieved to obtain the groundnut flour (Agriga & Iwe, 2009).

### 2.2 Determination of chemical composition of the products

All chemical analysis (proximate composition) of all samples was determined using AOAC, (2012).

### 2.3 Determination of functional properties of the blends

Bulk density (loose and packed) was determined according to the gravimetric method described by Mir et al. (2014). To determine the loose bulk density, 10 g of sample was measured into a calibrated 50 ml measuring cylinder with repeated mild tapping, until a constant volume was observed. The loose volume was recorded. For the determination of packed bulk density, the same sample was tapped inside the measuring flask with the aid of a rubber pad, More samples were added to make up to the graduated line before measurement were taken added to it up to the graduated line before weighing. The results were reported as g/ml.

$$\text{Bulk}\;\text{density}\;\left(g/ml\right)=\frac{\text{weight}\;\text{of}\;\text{sample}\;\text{(g)}}{\text{volume}\;\text{of}\;\text{sample}\;\text{(ml)}}$$

Water absorption capacity (WAC) was determined according to the method described by Adebowale, Adeyemi, and Oshodi (2005). Ten (10) ml of distilled water was added to 1 g of the sample in a beaker. The suspension was agitated using magnetic stirrer for 3 min. The suspension obtained was thereafter centrifuged at 2,058 × g for 30 min and the supernatant was measured into a 10 ml graduated cylinder. The absorbed water by the flour was considered as the change between the initial volume of the water and the volume of the supernatant. The water density was taken as 1.0 g/ml.

$$\text{WAC}=\frac{\text{weight}\;\text{of}\;\text{sample}\;\text{(g)}}{\text{volume}\;\text{of}\;\text{water}\;\text{used}-\text{volume}\;\text{of}\;\text{water}\;\text{unabsorbed}\;\text{(ml)}}\times 100$$

Oil absorption capacity (OAC) which is an index of the amount of oil retained within a protein matrix under certain conditions was determined according to the method described by Adebowale et al. (2005). About 10 ml of oil known specific gravity was added to 1 g of sample in a beaker. The suspension was stirred using magnetic stirrer for 3 min. The suspension obtained was thereafter centrifuged at 3500 rpm for 30 min and the supernatant was measured into a 10 ml granulated cylinder. The density of oil used was 0.931 g/ml. The change between the original volume of the oil and the volume of the supernatant was calculated as the oil absorbed by the flour.

$$\text{OAC}=\frac{\text{weight}\;\text{of}\;\text{sample}\;\text{(g)}}{\text{volume}\;\text{of}\;\text{oil}\;\text{used}-\text{volume}\;\text{of}\;\text{oil}\;\text{unabsorbed}\;\text{(ml)}}\times 100$$

Swelling capacity was determined according to the method of Okaka and Potter (1977). About 10 g of the sample was measured into a 100 ml graduated cylinder at room temperature; distilled water was added to give a total volume of 50 ml. The graduating cylinder was tightly covered at the top and mixed by inverting the cylinder repeatedly for 2 min, left to stand for another 8 min and the volume was recorded per gram of its original dry weight

$$\text{Swelling capacity (SC)}=\frac{\text{weight}\;\text{of}\;\text{wet}\;\text{sample}}{\text{weight}\;\text{of}\;\text{dry}\;\text{sample}}$$

Swelling index was determined according to the described method of Ukpabi and Ndimele (1990). 3 g of each flour sample was transferred into a 50 ml graduated cylinder. The flour samples were mildly leveled and the volume recorded. Distilled water (30 ml) was added to each sample and the cylinder was swirled and allowed to stand for 60 min before swelling level was observed. The swelling index was calculated as a multiple of the original volume.

$$\text{Swelling index (SI)}=\frac{\text{volume}\;\text{after}\;\text{soaking}-\text{volume}\;\text{before}\;\text{soaking}}{\text{Original}\;\text{weight}\;\text{of}\;\;\text{sample}}$$

Pasting properties were determined according to the method described by Ikegwu et al. (2009), using the rapid visco analyzer (RVA). A 3 g sample was weighed into 50 ml bottle containing distilled water with paddle placed inside canisters water. The flour samples were injected into the rapid visco analyzer at a heating temperature of 50°C for 1 min, 95°C for 3.8 min, and at 50°C for 1.4 min, the pasting results of the flour samples were recorded.

### 2.4 Physicochemical properties

The method of Benesi (2005) was employed in the determination of pH; A 5 g of samples was weighed in triplicates into different beakers and mixed with 20 ml of distilled water. The resulting suspension was stirred for 5 min and left to settle for 10 min. The pH of the liquid phase was measured using a calibrated pH meter.

### 2.5 Mineral analysis

Determination of mineral element was done by weighing 30 g of each sample using electric weighing balance. The sample was then ashed in a furnace at ashing temperature of 550°C. Afterward, 1.083 g was weighed and out of which 1 g of the sample was digested. The 1 g, part of the sample was placed into a beaker and 30 ml of nitric acid and distilled water was then added to the sample in the beaker. The sample was then warmed over water bath for 35 min and then allowed to cool. The digested sample was then filtered using Whatman filter paper and diluted with water to a volume of 100 ml. The sample was then run at a particular wavelength using the atomic absorption spectrophotometer to quantify the various mineral elements present in the sample.

### 2.6 Determination of Antinutritional contents

Determination of tannin was done by weighing 0.2 g of sample into a 50 ml bottle, with an addition of 10 ml of 70% aqueous acetone covering the sample. The samples were agitated in the shaker for 2 h at 30^°^C and the solutions centrifuged. While 0.2 ml of each solution and 0.8 ml of distilled water were transferred into test tubes, and 1 ml tannin acid standard was prepared by mixing 0.5 mg/ml stock solution with 0.5 ml distilled water. 0.5 ml of Folin ciocateau reagent was added to the samples and standard with the addition of 2.5 ml of 20% Na_2_CO_3_. The solutions were incubated at room temperature for 40 mins, whereas its absorbance was read at 725 nm against a blank reagent concentration of the same solution and a standard tannic acid curve was prepared (Makkar & Goodchild, 1996).

$$\text{Tannin(mg/g)}=\frac{\text{Abs.}\;\text{of}\;\text{sample}}{\text{Slope}\;\text{of}\;\text{standard}\;\text{curve}}$$

Phytate was determined according to the method of Wheeler and Ferrel (1971). 4 g Sample was soaked in 100 ml of 2% HCl for 3 h and then filtered through a No 1 Whatman filter paper. About 25 ml was taken out of the filtrate and placed inside a conical flask and 5 ml of 0.3% of ammonium thiocyanate solution was added as an indicator. After which 53.5 ml of distilled water was added to give it the proper acidity and this was titrated against 0.00566 g per milliliter of standard iron (iii) chloride solution that contained about 0.00195 g of iron per milliliter until a brownish yellow coloration persists for 5 min.

Phyate=titrevalueofsample×titrevalueofstandard

Oxalate determination was determined by soaking 1 g of the sample in 75 ml of 1.5 N H_2_SO_4_ for 1 h and then filtered through a No 1 Whatman filter paper. About 25 ml was taken out of the filtrate and placed inside a conical flask and this was titrated hot about (80–90°C) against 0.1 mol/L KMnO_4_ until a pink color that persists for 15 s (Day & Underwood, 1986).

### 2.7 Sensory analysis

The sensory evaluation was performed using the method of (Akinjaiyeju, 2009). Panelist of breast feeding mothers was used for the evaluation of aroma, appearance, taste, mouthfeel, consistency, and general acceptability. The scoring was based on a 9‐point hedonic scale, with results subjected to analysis of variance at 5% level of significance and means were separated using the Duncan Multiple Range Test.

### 2.8 Statistical analysis

The data generated were subjected to analysis of variance using the SPSS statistical package (10.00) 2000 edition. Significant of treatment means were tested at 5% level of probability using Duncan Multiple Range Test (DMRT).

## 3 RESULTS AND DISCUSSION

### 3.1 Proximate composition of formulated complementary foods

The proximate composition of the formulated complementary food samples from blends of sorghum, Irish potato, and groundnut flour is shown in Table 1. The moisture content of sprouted samples (SIGO, SIG1, and SIG2) ranged from 10.40% to 12.70% and was significantly different along the columns. Moisture content was highest in the sample (SIGO) 12.70% and lowest in (SIG2) 10.40%. The moisture content of unsprouted samples (NSIGO, NSIG1, and NSIG2) ranged from 8.44% to 11.20%, sample (NSIGO) was highest 11.20% and lowest in sample (NSIG1) 8.44%, Moisture content decreased with increased substitution of both sprouted and unsprouted complementary food from sorghum–Irish potato–groundnut. Sprouting increased the moisture content of the samples. This result was in conformity with Colmenares De Ruiz and Bressani (1990) who found a significant increase in moisture content level of Amaranth grain after sprouting for 48 h. High moisture content affects the storability of the complementary food (Udensi et al., 2012).

**Table 1:** Proximate compositions (g/100 g) of the formulated complementary Foods from sorghum, Irish potato, and groundnut

Samples	Moisture content (%)	Crude protein (%)	Total ash (%)	Crude fat (%)	Crude fiber (%)	Carbohydrate (%)
SIG0	12.70^a^ ± 0.05	8.22^e^ ± 0.03	1.76^a^ ± 0.06	3.31^d^ ± 0.01	6.65^c^ ± 0.02	67.50^b^ ± 0.20
SIG1	11.50^b^ ± 0.03	12.91^c^ ± 0.02	1.53^c^ ± 0.04	3.35^d^ ± 0.21	6.67^c^ ± 0.23	65.10^c^ ± 0.11
SIG2	10.40^c^ ± 0.06	14.53^a^ ± 0.1	1.62^b^ ± 0.03	3.47^c^ ± 0.01	6.75^b^ ± 0.32	66.65^d^ ± 0.03
NSIG0	11.20^b^ ± 0.10	7.40^f^ ± 0.10	1.77^a^ ± 0.02	3.56^c^ ± 0.07	6.77^b^ ± 0.02	69.15^a^ ± 0.09
NSIG1	8.44^d^ ± 0.36	11.40^d^ ± 0.20	1.65^b^ ± 0.01	3.61^b^ ± 0.05	6.88^a^ ± 0.03	65.55^c^ ± 0.01
NSIG2	10.60^c^ ± 0.10	13.30^b^ ± 0.15	1.62^b^ ± 0.16	3.73^a^ ± 0.02	6.85^a^ ± 0.02	62.94^e^ ± 0.02

SIG0 –100% Sprouted sorghum; SIG1–90% Sprouted, 5% Irish potato, and 5% groundnut; SIG2 – 80% Sprouted sorghum, 10% Irish potato, and 10% groundnut; NSIG0 – 100% unsprouted sorghum; NSIG1 – 90% unsprouted sorghum, 5% Irish potato, and 5% groundnut; NSIG2 – 80% unsprouted sorghum, 10% Irish potato, and 10% groundnut.

Means of triplicate determinations  ±  S.D with different superscripts on the same column are significantly different at (*p* ≤ .05).

The protein content of the sprouted samples ranged from 8.22% to 14.53% and values were significantly different along the column. Protein content increased with sprouting. Sample SIG2 had the highest protein content 14.53% and SIG0 8.22% had the lowest. Protein content in the unsprouted samples ranged from 7.40% to 13.30%. The highest value was obtained in (NSIG2) 13.30%, whereas protein content was lowest in (NSIGO) 7.40%. There was a significant difference between the sprouted and unsprouted sample (SIG2) 14.53%. The increase in the protein content might be as a result of the mobilization of stored nitrogen in sorghum aiding the sprouting. This observation is consistent with the findings of (Elkhalifa & Bernhardt, 2009) who reported a significant increase in protein content of complementary food formulated from sprouted sorghum and millet (Akinola et al., 2017). Also, the substitution of groundnut flour in the produced complementary food could have increased the protein content of the food. This finding agrees with that of Baba et al. (2012) who reported an increase in protein content of complementary food by substituting sorghum flour with groundnut flour.

Ash content ranged from 1.53% to 1.62% in the sprouted sorghum–Irish potato–groundnut flour samples and ranged from 1.62% to 1.77% in the unsprouted sorghum–Irish potato–groundnut flour samples. The (NSIG0) 1.77% samples had the highest crude ash content compared to the (SIGO) 1.76%. There was no significant difference in both the sprouted and the unsprouted samples. This is in conformity with Colmenares De Ruiz and Bressani (1990) who found no significant difference in ash content level of Amaranth grain after sprouting. The ash content value according to the standard recommended for complementary food is 2.5% (WHO/FAO, 2003). This shows that there is a significant difference between the values obtained and the standard recommended value.

Crude fiber ranged from 6.65% to 6.75% in the sprouted sorghum–Irish potato–groundnut flour samples; it was highest in (SIG2) 6.75 and lowest in (SIG0) 6.65. Unsprouted sorghum–Irish potato–groundnut flour ranged from 6.77% to 6.88%. There was no significant difference between SIG and NSIG. The recommended intake of dietary fiber varies depending on age and gender with a range from about 25 to 38 grams per day (Hermann, 2002). The dietary reference intake is 14 grams per 1,000 kilocalories. Also, crude fat ranged from 3.31% to 3.47% in the sprouted samples whereas in the unsprouted samples (3.56%–3.73%). Sprouting results in a slight decrease in the fat content as reported by Mubarak (2005). However, this may also affect the storage or shelf life of the formulated food samples due to oxidative activities of fats (Fasasi, 2009).

Carbohydrate content ranged from 65.10% to 67.50% in sprouted sorghum–Irish potato–groundnut flour samples, it was highest in (SIGO) 67.50 and lowest in (SIG2) 66.65. Unsprouted sorghum–Irish potato–groundnut flour samples ranged from 62.94% to 69.15% and it was highest in (NSIG0) 69.15 and lowest in (NSIG2) 62.94. There was a significant difference between sprouted sorghum–Irish potato–groundnut flour samples and unsprouted sorghum–Irish potato–groundnut flour samples. Similar results were reported by Elemo et al. (2011) who reported 63.7%–77.4% of carbohydrate content in complementary food processed from sorghum and cowpea. The carbohydrate content of the produced complementary food met the recommended standard by WHO/FAO (2003) in the complementary food (≥65 g/100 g).

Moisture content, ash content and carbohydrate content decrease with the substitution of Irish potato and groundnut, whereas protein content, crude fat, and crude fiber content increase with the substitution of Irish potato and groundnut flour.

### 3.2 Mineral contents

Table 2 shows the mineral contents of the formulated complementary foods from the blends of sorghum, Irish potato, and groundnut flour samples. Calcium ranged from 101.33 to 121.33 g/100 g in unsprouted sorghum–Irish potato–groundnut flour samples and ranged from 91.00 to 103.00 g/100 g/100 g in the sprouted sorghum–Irish potato–groundnut flour samples. Potassium ranged from 535.00 to 766.67 g/100 g in the sprouted sorghum–Irish potato–groundnut flour samples and ranged from 106.00 to 956.00 g/100 g in the unsprouted sorghum–Irish potato–groundnut flour samples; sodium ranged from (25.00 to 108.00 g/100 g) in the sprouted sorghum–Irish potato–groundnut flour samples and ranged from (67.33 to 106.00 g/100 g) in the unsprouted sorghum–Irish potato–groundnut flour samples; and iron ranged from 6.60 to 7.60 g/100 g in sprouted sorghum–Irish potato–groundnut flour samples and ranged from 8.70 to 8.73 g/100 g in the nonsprouted sorghum–Irish potato–groundnut flour samples. Copper ranged from 0.80 to 1.29 g/100 g in the sprouted sorghum–Irish potato–groundnut flour samples and ranged from 0.60 to 0.70 g/100 g in the unsprouted sorghum–Irish potato–groundnut flour samples. Sprouted samples are significantly lower compared to the unsprouted samples this could be as a result of soaking during processing. Potassium is an essential nutrient needed for maintenance of total body fluid volume, acid and electrolyte balance, and normal cell function (Young, 2001). Effect of increased potassium intake result into blood lipids and other possible adverse effect (WHO, 2012), Calcium is an essential nutrient in the mineralization of bones and teeth and for regulating intracellular event in body tissues. It plays a role in muscle contraction and nerve function (Deborah, 2007). Inadequate calcium combined with adequate energy and protein intake may result in low calcium content of bone, which has implications for bone health later in life (Deborah, 2007). Iron is essential for the function of mammalian cells and development of the central nervous system, Iron is required for the production of red blood cells, transportation of oxygen from the lungs through the arteries to all cells throughout the body (Beard & Dawson, 1997). Iron deficiency in infants causes anemia, which occurs when stored iron is exhausted in the body and its metabolic demands are not met (WHO, 2016). They function in the synthesis of hemoglobin and myoglobin; Copper has an antioxidant role that protects cell‐free radical injury (Voskaki et al., 2010). It also contributes to the formation of ceruloplasmin. Deficiency of copper in infants leads to anemia, neutropenia, impartment of growth, abnormalities in glucose, and cholesterol metabolism (Shazia et al., 2012). Sodium is the principal cation in extracellular fluid in the body and is an essential nutrient necessary for the maintenance of plasma volume, acid‐base balance, transmission of nerve impulses, and normal cell function. Increased sodium consumption is associated with hypernatremia (Verbalis et al., 2010). Calcium, copper, iron, potassium, and sodium decreased with sprouting, the higher the substitution of Irish potato flour samples, the higher the mineral content in the complementary food samples.

**Table 2:** Mineral content of flours and formulated complementary foods from sorghum, Irish potato, and groundnut

Samples	Ca (mg/100 g)	Cu mg/100 g	Fe mg/100 g	Na mg/100 g	K mg/100 g
SIG0	91.00^d^ ± 1.53	0.60^d^ ± 0.21	7.60^b^ ± 0.10	25.00^e^ ± 1.00	487.33^d^ ± 1.29
SIG1	121.33^a^ ± 0.27	0.65^d^ ± 0.05	6.60^c^ ± 0.15	108.00^a^ ± 0.75	106.00^e^ ± 1.00
SIG2	120.50^b^ ± 0.50	0.70^c^ ± 0.15	6.60^c^ ± 0.20	63.30^d^ ± 1.03	766.67^b^ ± 2.25
NSIG0	101.33^c^ ± 0.25	0.80^b^ ± 0.10	8.73^a^ ± 0.15	67.33^c^ ± 1.53	535.00^c^ ± 1.25
NSIG1	121.33^a^ ± 0.27	1.27^a^ ± 0.15	7.73^b^ ± 0.19	86.00^b^ ± 1.25	751.33^b^ ± 1.53
NSIG2	120.50^b^ ± 0.50	1.29^a^ ± 0.25	8.70^a^ ± 0.10	106.00^a^ ± 1.00	956.67^a^ ± 1.00

Ca – Calcium, Cu – Copper, Fe – Iron, Na – Sodium, K – Potassium, SIG0 – 100% Sprouted sorghum; SIG1 – 90% Sprouted, 5% Irish potato, and 5% groundnut; SIG2 – 80% Sprouted sorghum, 10% Irish potato, and 10% groundnut; NSIG0 – 100% unsprouted sorghum; NSIG1 – 90% unsprouted sorghum, 5% Irish potato, and 5% groundnut; NSIG2 – 80% unsprouted sorghum, 10% Irish potato, and 10% groundnut.

Means of triplicate determinations  ±  S.D with different superscripts on the same column are Significantly different at (*p* ≤ .05).

### 3.3 Antinutritional contents

Table 3 shows the antinutritional contents in the fortified sprouted samples (SIG0, SIG1, SIG2), and unsprouted food samples (NSIG1 and NSIG2 and NSIG0). The tannin, oxalate, and phytate content of the sprouted samples ranged from 0.11 to 0.15 mg/100 g, 0.36 to 0.64 mg/100 g, and 6.32 to 6.34 mg/100 g, respectively, and for unsprouted samples, it ranged from 0.11 to 0.64 mg/100 g, 0.72 to 0.98, and 4.98 to 7.42 mg/100 g, respectively. Sprouting decreased the tannin, phytate, and oxalate when compared with the unsprouted sorghum–Irish potato–groundnut samples this could be a result of formation of hydrophobic association of tannins, phytate, and oxalate during sprouting Also, may be due to leaching of tannins, phytate, and oxalate into the water (Afam et al., 2016). However, the implication of high content of tannin is that it decreases the bioavailability of protein and causes a change to the gastrointestinal tract (GIT) (Afam et al., 2016). Antinutrient reduces with sprouting and with the substitution of Irish potato and groundnut.

**Table 3:** Antinutritional factors of the formulated complementary foods from Sorghum, Irish potato, and groundnut

Samples	Tannin (mg/100 g)	Oxalate (mg/100 g)	Phytate (mg/100 g)
SIG0	0.13^e^ ± 0.03	0.64^d^ ± 0.05	6.69^b^ ± 0.02
SIG1	0.11^c^ ± 0.02	0.45^e^ ± 0.04	6.32^c^ ± 0.03
SIG2	0.15^d^ ± 0.01	0.36^f^ ± 003	6.34^c^ ± 0.01
NSIG0	0.64^a^ ± 0.08	0.98^a^ ± 0.04	7.42^a^ ± 0.15
NSIG1	0.17^b^ ± 0.06	0.90^b^ ± 0.03	4.98^e^ ± 0.05
NSIG2	0.11^c^ ± 0.02	0.72^c^ ± 0.07	5.37^d^ ± 0.03

SIG0 –100% Sprouted sorghum; SIG1 – 90% Sprouted, 5% Irish potato, and 5% groundnut; SIG2 – 80% Sprouted sorghum, 10% Irish potato, and 10% groundnut; NSIG0 – 100% unsprouted sorghum; NSIG1 – 90% unsprouted sorghum, 5% Irish potato, and 5% groundnut; NSIG2 – 80% unsprouted sorghum, 10% Irish potato, and 10% groundnut.

Means of triplicate determinations  ±  S.D with different superscripts on the same column are significantly different at (*p* ≤ .05).

### 3.4 Functional properties

Table 4 shows that the packed bulk density (PBD) of complementary food made from sprouted sorghum–Irish potato–groundnut flour ranged from 0.84 to 0.86 g/ml, whereas the unsprouted samples (0.86 to 0.89 g/ml). NSIG1 (0.89 g/ml) had the highest Packed bulk density, whereas SIG2 (0.86 g/ml) had the highest among the sprouted sorghum–Irish potato–groundnut samples. Also, the loosed bulk density (LBD) ranged from 0.41 to 0.46 g/ml in sprouted sorghum–Irish potato–groundnut samples and unsprouted sorghum–Irish potato–groundnut (0.60–0.63 g/ml). Sample NSIG2 had the highest loosed bulked density (0.63 g/ml). The values obtained for both sprouted and unsprouted sorghum–Irish potato–groundnut complementary foods were within the standard stipulated by FAO/WHO (0.7 g/ml). However, similar values had been reported in complementary foods by Ikujenlola & Fashakin, (2005) and Osundahunsi & Aworh (2002).

**Table 4:** Functional properties of the formulated complementary foods from Sorghum, Irish potato, and groundnut

Samples	BD Pack (g/ml)	BD Loose (g/ml)	WAC (mg/g)	OAC (mg/ml)	SC (mg/g)	SI (mg/g)
SIG0	0.84^a^ ± 0.01	0.41^b^ ± 0.02	0.77^a^ ± 0.02	0.61^b^ ± 0.02	1.26^a^ ± 0.05	0.48^b^ ± 0.02
SIG1`	0.85^a^ ± 0.03	0.44^b^ ± 0.04	0.67^a^ ± 0.25	0.58^c^ ± 0.01	1.23^a^ ± 0.21	0.38^c^ ± 0.09
SIG2	0.86^a^ ± 0.06	0.46^b^ ± 0.01	0.68^a^ ± 0.01	0.67^a^ ± 0.02	1.24^a^ ± 0.01	0.36^c^ ± 0.01
NSIG0	0.86^a^ ± 0.01	0.60^a^ ± 0.12	0.85^a^ ± 0.25	0.64^b^ ± 0.01	1.26^a^ ± 0.09	0.55^a^ ± 0.01
NSIG1	0.89^a^ ± 0.71	0.61^a^ ± 0.12	0.82^b^ ± 0.02	0.69^a^ ± 0.01	1.25^a^ ± 0.05	0.42^b^ ± 0.01
NSIG2	0.87^a^ ± 0.01	0.63^a^ ± 0.21	0.83^b^ ± 0.30	0.44^d^ ± 0.02	1.25^a^ ± 0.02	0.47^b^ ± 0.01

BD – bulk density, WAC – water absorption capacity, OAC – oil absorption capacity, SC – swelling capacity, SI – solubility Index, SIG0 – 100% Sprouted sorghum, SIG1 – 90% Sprouted, 5%Irish potato, and 5% groundnut, SIG2 – 80% sprouted sorghum, 10% Irish potato, and 10% groundnut, NSIG0 – 100% unsprouted sorghum, NSIG1 – 90% unsprouted sorghum, 5% Irish potato, and 5% groundnut, NSIG2 – 80% unsprouted sorghum, 10% Irish potato, and 10% groundnut.

Means with different superscripts on the same column are significantly different at *p* ≤ .05.

The water absorption capacity (WAC) is important in the development of ready to eat foods, and a high WAC may predict product cohesiveness (Oduro et al., 2000). WAC of samples SGI0, SGI1, SGI2 decreased with Irish potato and groundnut flour substitution, whereas a slight increase was obtained in samples NSIG0, NSIG1, and NSIG2. The substitution of Irish potato and groundnut flour improves the textural ability of the complementary foods. This supports the findings of Oduro et al. (2000) in a study on sorghum‐pigeon pea complementary food.

The swelling capacity ranged from 1.26 to 1.23 in the sprouted sorghum–Irish potato–groundnut flour samples and was highest in SIG0; it ranged from 1.26 to 1.25 in the unsprouted sorghum–Irish potato–groundnut flour samples and was highest in NSIG0. There was a slight decrease in each sample. This could be as a result of the swelling of the starch granules which leads to disruption of some of the intermolecular hydrogen bonds thus allowing more water to enlarge and enter the granules (Ihekoronye & Ngoddy, 1985).

Oil absorption capacity ranged from 0.67 to 0.58 in the sprouted sorghum–Irish potato–groundnut flour sample. It was highest in sample (NSIG2); it ranged from 0.69 to 0.44 in the unsprouted sorghum–Irish potato–groundnut flour sample, whereas sample NSIG1 had the highest value. There were no significant differences between the sprouted and unsprouted sorghum–Irish potato–groundnut flour samples. Oil absorption capacity is important since oil acts as flavor retainer and increases the palatability of foods (Wang & Kinsella, 1976).

Solubility index ranged from (0.36 to 0.48 mg/g) in the sprouted sorghum–Irish potato–groundnut flour samples and (0.42–0.55 mg/g) in the unsprouted sorghum–Irish potato–groundnut flour samples. Solubility index increases with sprouting and there were significant differences between each sample. The lowest solubility index was obtained in sample SIG2, this indicate that the water occupied a small volume which was relatively low when compared with other samples (Brou et al., 2013).

**Figure 1:**
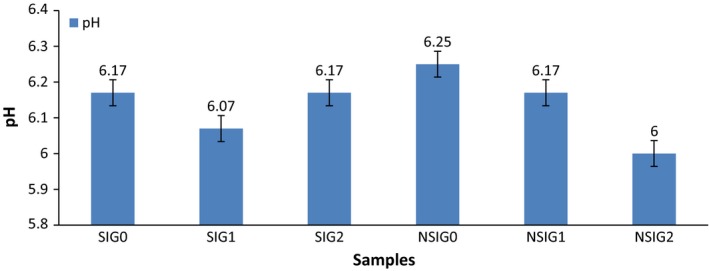
*p*^H^ values of the formulated food samples from sorghum–Irish potato–groundnut; Keys SIG0 – 100% Sprouted sorghum; SIG1 – 90% Sprouted, 5% Irish potato, and 5 % groundnut; SIG2 – 80% sprouted sorghum, 10% Irish potato, and 10% groundnut; NSIG0 – 100% unsprouted sorghum; NSIG1 – 90 % unsprouted sorghum, 5% Irish potato, and 5% groundnut; NSIG2 – 80 % unsprouted sorghum, 10% Irish potato, and 10% groundnut

Figure 1 shows the *p* ^*H*^ Values of the Formulated Food Samples from Sorghum, Irish potato and Groundnut. The pH value ranged from 6.07 to 6.17 in the sprouted sorghum–Irish potato–groundnut samples, it was highest in (SIG2) 6.17 and lowest in sample the (SIG1) 6.07. Unsprouted sorghum–Irish potato–groundnut flour samples ranged from (6.00 to 6.25) it was highest in NSIG0 (6.25) and lowest in NSIG2 (6.0). There was no significant difference between the sprouted and unsprouted samples. The pH values (6.00–6.25) of the formulated Complementary foods were within the range (5.60–6.7) reported by Adenekan and Oyewole (2010) for “*Ogi”* produced from germinated sorghum supplemented with soybeans. pH decreases with sprouting and increases with unsprouted. Also, pH decreases with increase in substitution for the sprouted sorghum–Irish potato–groundnut flour samples and increases with substitution in unsprouted sorghum–Irish potato–groundnut flour samples.

### 3.5 Pasting properties

Peak viscosity is a measure of the ability of starch to form a paste on cooking. The peak viscosity ranged from (43.08 to 23.57 RVU) in both sprouted and unsprouted sorghum–Irish potato–groundnut flour samples is shown in Table 5. Peak viscosity was lower in the unsprouted sorghum–Irish potato flour samples when compared with the sprouted sorghum–Irish potato flour samples. There were significant differences between the sprouted and unsprouted sorghum–Irish potato–groundnut flour samples; this could be as a result of the activity of amylase enzymes developed during sprouting process which degrades the starch to simpler units (Fagbemi, 2007). The reduction in viscosity of the diets is advantageous, the gruel prepared from it would be watery and more solid could be added; this will amount into adding more nutrients and energy which is better for the growing children. The difference in the peak viscosity of the sprouted sorghum–Irish potato–groundnut flour samples and unsprouted sorghum–Irish potato–groundnut flour samples indicates that there were differences in the rate of water absorption and starch granule swelling during heating (Ragaee & Abdel‐Aal, 2006).

**Table 5:** Pasting properties of the formulated complementary foods from sorghum, Irish potato, and groundnut

Samples	Peak Visc. (RVU)	Trough (RVU)	Breakdown (RVU)	Final Visc (RVU)	Setback (RVU)	Peak Time (Min)	PastingTemp. (°C)
SIG0	43.08^a^	41.08^a^	24.00^c^	52.43^d^	59.00^a^	7.00^a^	87.25^a^
SIG1	35.41^b^	33.33^b^	25.00^c^	53.56^d^	45.41^b^	7.00^a^	88.05^a^
SIG2	32.00^b^	29.33^b^	32.00^b^	60.67^c^	41.33^cb^	7.00^a^	88.75^a^
NSIG0	40.75^a^	35.66^b^	61.00^a^	84.33^a^	48.67^b^	5.07^a^	86.35^a^
NSIG1	23.58^c^	22.58^c^	12.00^d^	72.42^b^	47.10^b^	7.00^a^	88.90^a^
NSIG2	23.67^c^	22.50^c^	14.00^d^	69.33^b^	46.00^b^	7.00^a^	89.60^a^

SIG0 –100% Sprouted sorghum; SIG1 – 90% Sprouted, 5% Irish potato, and 5% groundnut; SIG2 – 80% Sprouted sorghum, 10% Irish potato, and 10% groundnut; NSIG0 – 100% unsprouted sorghum; NSIG1 – 90% unsprouted sorghum, 5% Irish potato, and 5% groundnut; NSIG2 – 80% unsprouted sorghum, 10% Irish potato, and 10% groundnut.

Means of triplicate determinations  ±  S.D with different superscripts on the same column are significantly different at (*p* ≤ .05).

Trough is the minimum viscosity which measures the ability of paste to withstand breakdown during cooling. The trough value ranged from (41.08 to 22.50 RVU) in the sprouted and unsprouted sorghum–Irish potato–groundnut flour. There were significant differences between the sprouted sorghum–Irish potato–groundnut flour when compared with the unsprouted sorghum–Irish potato–groundnut flour samples.

The breakdown viscosity of the weaning food blends ranged between (61 and 14) in the sprouted and unsprouted sorghum–Irish potato–groundnut flour with sample NSIG0 having the highest value. The breakdown viscosity is essentially a measure of the degree of paste stability or starch granule disintegration during heating (Dengate, 1984). Therefore, the complementary food blends from the sample with the lowest breakdown viscosity will have a more stable paste during heating than others with higher breakdown viscosity (Farhat et al., 1999). However, sprouting increased the breakdown viscosity thereby making the paste less stable during heating. This period is commonly associated with a breakdown in viscosity. The ability of starch to withstand heating at high temperature and shear stress is an important factor in many processes. Elofsson et al. (1997) noted that gel formation of proteins is the result of a two‐step process involving, first, the partial denaturation of individual proteins to allow more access to the reactive side groups within the protein molecules and second aggregation of these proteins by means of reactive side groups into a continuous three‐dimensional network structure capable of retaining significant amount of water and also exhibiting same structural rigidity. This phenomenon is of importance in foods since it contributes significantly to the textural and rheological properties of various foods.

The final viscosity is an important parameter in predicting and defining the final textural quality of food in terms of its hardness and elasticity. The final viscosity ranged between (84.33 and 52.53 RVU) in both sprouted and unsprouted–Irish potato–groundnut flour samples The inclusion of sprouted sorghum flour in the complementary food blends was observed to cause a general reduction in the final viscosity. This observation may be attributed to the enzymatic activity that had occurred during the sprouting process whereby the starch molecules were degraded (Xu et al., 2012). Consequently, the degraded starch structure (particularly amylose structure) resulted in reduced final viscosity due to minimized aggregation of the amylose molecules in the gelatinized paste during cooling (Chung et al., 2012). It had also been observed that the exhibition of final viscosity in a gelatinized paste is as a result of the aggregation of the amylose molecules in the paste (Farhat et al., 1999). Since the viscosity of infants complementary food plays an important role in the food acceptability as well as on infants’ energy intake (Treche & Mbome, 1999), the inference that can be made from these observations is that the complementary food blends from samples SIG0 and SIG1 which exhibited relatively low final viscosity values, might be the most appropriate for developing weaning foods. Final viscosity is usually regarded as an indicator of the stability of the cooked paste in actual use (Ragaee & Abdel‐Aal, 2006).

The setback viscosity of the complementary food samples ranged from (41.33 to 89.00 RVU) in the sprouted and unsprouted sorghum–Irish potato–groundnut flour samples. This phase is commonly described as the setback region and is related to retrogradation and reordering of starch molecules. The setback viscosity is usually regarded as an index of retrogradation tendency of the paste prepared from a starchy food (Sandhu & Singh, 2007) and the higher the value, the greater the retrogradation tendency. However, sprouting in SIG flour samples tends to reduce the setback viscosity when compared with NSIG flour samples. Ragaee and Abdel‐Aal (2006) had earlier reported that low setback values indicate the low rate of starch retrogradation and syneresis. Therefore, the observed variation in setback values has a strong implication on the variability in retrogradation tendency of the complementary food.

The pasting temperature of the weaning food blends ranged between 87.25 and 89.60°C with SIG0 and NSIG2 having the lowest and highest values, respectively (*p* ≤ .05). Sprouted sorghum–Irish–potato flour samples had a reduction in the pasting temperatures compared to the unsprouted sorghum–Irish potato–groundnut flour samples. There were no significant differences between the pasting temperatures of the complementary food samples. This indicates that the samples exhibited the same gelatinization temperatures. The pasting temperature provides an indication of the minimum temperature required to cook a given sample, which can also have implications on energy usage (Ragaee & Abdel‐Aal, 2006). The peak time of the weaning food blends ranged between 5.07 and 7.00. NSIG0 had the lowest value, whereas other samples were not significantly different. The peak time is usually regarded as an indication of the total time taken by each blend to attain its respective peak viscosity. Thus, weaning food blends with a lower peak time will cook faster than that with a higher peak time.

### 3.6 Sensory evaluation

The sensory scores of the complementary foods as shown in Table 6 revealed that significant difference exists in terms of aroma, texture, taste, and general acceptability in all the parameters evaluated. The control sample was more accepted followed by SIG2, SIG1, SIG0, NSIG0, NSIG2, and NSIG1.

**Table 6:** Sensory properties of the formulated complementary foods from sorghum, Irish potato, and groundnut

Samples	Appearance	Aroma	Taste	Mouthfeel	Consistency	Acceptability
Control	8.00^a^ ± 0.32	7.60^a^ ± 0.75	7.50^a^ ± 0.83	6.60^a^ ± 1.50	5.40^a^ ± 1.64	7.40^a^ ± 0.88
SIG0	6.30^b^ ± 1.38	5.45^d^ ± 1.47	5.90^b^ ± 1.89	6.05^b^ ± 1.27	5.20^a^ ± 1.36	5.75^d^ ± 1.08
SIG1	6.65^b^ ± 1.31	6.45^b^ ± 1.01	5.90^b^ ± 1.33	5.65^d^ ± 1.23	4.75^b^ ± 1.12	6.20^c^ ± 1.00
SIG2	5.55^d^ ± 1.79	5.15^e^ ± 1.76	5.55^d^ ± 1.40	4.65^e^ ± 1.79	5.05^b^ ± 1.82	6.70^b^ ± 1.12
NSIG0	5.90^c^ ± 1.21	5.25^e^ ± 1.29	4.70^e^ ± 1.59	5.80^c^ ± 1.74	4.40^d^ ± 2.21	4.95^e^ ± 1.15
NSIG1	5.65^d^ ± 1.73	5.50^d^ ± 1.36	4.90^e^ ± 1.59	4.35^f^ ± 1.39	4.90^c^ ± 2.6	4.00^f^ ± 1.12
NSIG2	5.80^c^ ± 2.25	5.80^c^ ± 1.234	5.70^c^ ± 1.59	5.60^d^ ± 1.76	3.90^e^ ± 1.52	4.15^g^ ± 1.35

SIG0 – 100% Sprouted sorghum; SIG1 – 90% Sprouted, 5% Irish potato, and 5% groundnut; SIG2 – 80% Sprouted sorghum, 10% Irish potato, and 10% groundnut; NSIG0 – 100% unsprouted sorghum; NSIG1 – 90% unsprouted sorghum, 5% Irish potato, and 5% groundnut; NSIG2 – 80% unsprouted sorghum, 10% Irish potato, and 10% groundnut.

Means of triplicate determinations  ±  S.D with different superscripts on the same column are significantly different at (*p* ≤ .05).

## 4 CONCLUSION

The study showed that complementary food products formulated from locally available food commodities (sorghum, Irish potato, and groundnut) met the macro nutritional needs of children between 6 months and 2 years. On the other hand, the formulated local complementary diets did not meet some of the recommended micronutrient (minerals) requirements of infants and children in some area. Therefore, further investigations on fortification with appropriate micronutrients or micronutrients‐dense food stuff should be carried out. Also, this study established that the processing methods (sprouting) carried out had a positive influence on the nutritive values of the formulated diets.

## CONFLICT OF INTEREST

None declared.

## References

[fsn3556-bib-0001] Adebowale, Y. A. , Adeyemi, I. A. , & Oshodi, A. A. (2005). Functional and physicochemical properties of flour of six *Mucuna* species. African Journal of Biotechnology, 4(12), 1461–1468.

[fsn3556-bib-0002] Adeleke, R. O. , & Adedeji, J. O. (2012). Functional properties of wheat and sweet potato flour blends. Pakistan Journal of Nutrition, 9(6), 535–538.

[fsn3556-bib-0003] Adenekan, A. O. , & Oyewole, O. B. (2010). Production of ‘Ogi’ from germinated sorghum supplemented with soybeans. African Journal of Biotechnology, 9, 7114–7121.

[fsn3556-bib-0004] Afam, O. C. , Agugo, U. A. , & Anyaegbu, E. C. (2016). Effect of germination on the nutritional and anti‐nutritional contents of mung bean (*Vignradiata)* . African Journal of Agricultural Science and Technology, 4(7), 801–805.

[fsn3556-bib-0005] Agriga, A. N. , & Iwe, M. O. (2009). Proximate composition of cookies from cassava groundnut – corn starch blends. Nigerian Food Journal, 27, 102–107.

[fsn3556-bib-0006] Akinjaiyeju, O. (2009). Quality Control for the food industry. A statistical approach: Concept publications.

[fsn3556-bib-1000] Akinola, S. A. , Badejo, A. A. , Osundahunsi, O. F. , & Edema, M. O. (2017). Effect of preprocessing techniques on pearl millet flour and changes in technological properties. International Journal of Food Science & Technology, 52(4), 992–999.

[fsn3556-bib-0007] AOAC (2012). Official methods of analysis (19th edn). Gaithersburg M. D., USA: Association of Official Analytical Chemists

[fsn3556-bib-0008] Asibuo, J. Y. , Arkromah, R. , Safo‐Kantanka, O. , & Adu‐Dapaah, H. K . (2008). Evaluation of Nutritional Quality of Groundnut (*Arachis hypogaea* L.) from Ghana. African Journal of Food Agriculture, Nutrition and Development 8(2):134–141. https://doi.org/10.4314/ajfand.v812.19185.

[fsn3556-bib-0009] Baba, G. M. , Modus, S. , Falmata, A. S. , Hajjagannah, L. , & Ibrahim, Z. (2012). Evaluation of the nutritional value of sprouted sorghum fortified with cowpea and groundnut. Journal of Agricultural Science., 2(11), 292–296.

[fsn3556-bib-0010] Beard, J. L. , & Dawson, H. D. (1997). Iron In O'Dell B. L., & Sunde R. A. (Eds.), Handbook of Nutritionally Essential Mineral Elements (pp. 275–334). New York: CRC Press.

[fsn3556-bib-0011] Benesi, I. R . (2005). Characterisation of Malawian cassava germplasm for diversity, starch extraction and its native and modified properties, PhD Thesis, Department of Plant Sciences, University of the Free State, South Africa p. 74‐123.

[fsn3556-bib-0012] Bolarinwa, I. F. , Olaniyan, S. A. , Adebayo, L. O. , & Ademola, A. A. (2015). Malted sorghum‐soy composite flour: Preparation, chemical and physico‐chemical properties. Journal of Food Process and Technology, 6(8), https://doi.org/10.4172/2157-7110.1000467

[fsn3556-bib-0013] Brou, K. , N'Da‐Kouassi, A. M. , Kouadio, J. H. , Guehi, T. , N'Guessan, K. F. , & Gnakri, D. (2013). Biochemical characterization and functional properties of weaning food made from cereals (millet, maize) and legumes (beans, soybeans). Journal of Food Chemistry and Nutrition, 01(01), 22–32.

[fsn3556-bib-0014] Chung, H. , Cho, D. , Park, J. , Kweon, D. , & Lim, S. (2012). In vitro starch digestibility and pasting properties of germinated brown rice after hydrothermal treatments. Journal of Cereal Science, 56, 451–456. https://doi.org/10.1016/j.jcs.2012.03.010

[fsn3556-bib-0015] Colmenares De Ruiz, A. S. , & Bressani, R. (1990). Effect of germination on the chemical composition and nutritive value of amaranth grain. Cereal Chemistry, 67(6), 519–522.

[fsn3556-bib-0016] Day, R. A. , & Underwood, A. L. (1986). Quantitive analysis, 5th ed. (p. 701). Prentice: Hall publication p.

[fsn3556-bib-0017] Deborah, A. S. (2007). Calcium supplementation in clinical practice: A review of forms. Doses and Indications, Nutrition in Clinical Practice, 22, 286–296. 17507729 10.1177/0115426507022003286

[fsn3556-bib-0018] Dengate, H. N. (1984). Swelling, pasting and gelling of wheat starch In Pomeranz Y. (Ed.), Advances in cereal science and technology (pp. 49–82). USA: American Association of Cereal Chemists.

[fsn3556-bib-0019] Elemo, G. N. , Elemo, B. O. , & Okafor, J. N. C. (2011). Preparation and Nutritional Composition of a Weaning Food Formulated from Germinated Sorghum (*Sorghum bicolor*) and Steamed Cooked Cowpea (*Vigna unguiculata* Walp.). American Journal of Food Technology, 6(5), 413–421. https://doi.org/10.3923/ajft.2011.413.421

[fsn3556-bib-0020] Elkhalifa, A. O. , & Bernhardt, R. (2009). Influence of grain germination on functional properties of sorghum flour. Food Chemistry, 121, 387–392. https://doi.org/10.1016/j.foodchem.2009.12.041

[fsn3556-bib-0021] Elofsson, C. , Dejmek, P. , Paulson, M. , & Burling, H. (1997). Characterization of a cold gelling whey concentrate. International Dairy Journal, 7, 601–607. https://doi.org/10.1016/S0958-6946(97)00050-2

[fsn3556-bib-0022] Eshun, G. , Adu‐Amankwah, E. , & Barimah, J. (2013). Nutrients content and lipid characterization of seed pastes of four selected peanut (*Arachishypogaea*) varieties from Ghana. African Journal of Food Science, 7(10), 375–381. https://doi.org/10.5897/AJFS12.136

[fsn3556-bib-0023] Fagbemi, T. N. (2007). Effects of processing on the nutritional composition of flute pumpkin (*Telfaria occidentalis*) seed flours. Nigerian Food Journal, 25(1), 1–22.

[fsn3556-bib-0024] FAO and ICRISAT . (1996). The World Sorghum Economies; Facts, Trends and Outlook. Rome, Italy and Andhra Pradesh, India: FAO and ICRISAT.

[fsn3556-bib-0025] Farhat, I. A. , Oguntona, T. , & Neale, J. R. (1999). Characterisation of starches from West African yams. Journal of the Science of Food and Agriculture, 79, 2105–2112. https://doi.org/10.1002/(ISSN)1097-0010

[fsn3556-bib-0026] Fasasi, O. S. (2009). Proximate, Antinutritional factors and functional properties of processed pearl millet (*Pennisetum glaucum*). Journal of Food Technology, 7(3), 92–97.

[fsn3556-bib-0027] Habtamu, F. G. , & Negussie, R. (2014). Antinutritional factors in plant foods: Potential health benefits and adverse effects. International Journal of Nutrition and Food Sciences., 3(4), 284–289. https://doi.org/10.11648/j.ijnfs.20140304.18

[fsn3556-bib-0028] Hamilton, C. , Andy, A. , & Dave, W . (2004). Importance and usefulness of potato In Advance Agriculture Science (2nd edition) (pp. 614–625). New York/London: Plenum Publisher.

[fsn3556-bib-0029] Hermann, J. J . (2002). Dietary Fiber T‐3138. Stillwater: Oklahoma Cooperative Extension Service.

[fsn3556-bib-0030] Ihekoronye, I. A. , & Ngoddy, P. O. (1985). Integrated food science and technology for the tropics. London: Macmillian Publishers Ltd.

[fsn3556-bib-0031] Ikanone, C. E. O. , & Oyekan, P. O. (2014). Effect of boiling and frying on the total carbohydrate, Vitamin C and Mineral Contents of Irish *(Solanun tuberosum)* and Sweet *(Ipomea batatas)* Potato Tubers. Nigerian Food Journal, 32(2), 33–39. https://doi.org/10.1016/S0189-7241(15)30115-6

[fsn3556-bib-0032] Ikegwu, O. J. , Nwobasi, V. N. , Odoh, M. O. , & Oledinma, N. U. (2009). Evaluation of the pasting and some functional properties of starch isolated from some improved cassava varieties in Nigeria. African Journal of Biotechnology, 8(10), 2310–2315.

[fsn3556-bib-0033] Ikujenlola, A. V. , & Fashakin, J. B. (2005). Bioassay assessment of a complementary diet prepared from vegetable proteins. Journal of Food Agriculture and Environment, 3(3), 20–22.

[fsn3556-bib-0034] Kleinman, R. E . (2004). Complementary feeding. Pediatric Nutrition Handbook. (5^th^ Ed). ELK Grove Village, IL:AAP. p: 103‐115.

[fsn3556-bib-0035] Makkar, A. O. S. , & Goodchild, A. V . (1996). Qualification of tannins: A laboratory manual. International Centre for Agriculture Research in the Dry Areas (ICARDA):Aleppo, Syria iv + 25 pp.

[fsn3556-bib-0036] Miles, M. J. , Morris, V. J. , Orford, P. D. , & Ring, S. G. (1985). The roles of amylose and amylopectin in the gelation and retrogradation of starch. Carbohydrate Research, 135, 271 https://doi.org/10.1016/S0008-6215(00)90778-X

[fsn3556-bib-0037] Mir, N. A. , Gul, K. , & Riar, C. S. (2014). Physicochemical, pasting and thermal properties of water chestnut flours: A comparative analysis of two geographic sources. Journal of Food Processing & Preservation, https://doi.org/10.1111/jfpp.12359

[fsn3556-bib-0038] Modu, S. , Ibrahim, Z. , Hajjagana, L. , Falmata, A. S. , Babagana, M. , & Bintu, B. P . (2013). Production and evaluation of weaning meal from fermented red maize fortified with cowpea. Academia Journal of Food Research 1(3):050–058.https://doi.org/10.15413/ajfr.2012.0117

[fsn3556-bib-0039] Mubarak, A. E. (2005). Nutritional composition and Antinutritional factors of mung bean seeds (*Phaseolus aureus*) as affected by some home traditional processes. Food Chemistry, 89(4), 489–495. https://doi.org/10.1016/j.foodchem.2004.01.007

[fsn3556-bib-0040] Oduro, I. , Ellis, W. O. , Aryeetey, S. K. , Ahenkora, K. , & Otoo, J. A. (2000). Pasting characteristics of starch from new varieties of sweet potato. Journal Tropical Science, 40, 25–28.

[fsn3556-bib-0041] Okaka, J. C. , & Potter, N. N. (1977). Functional and storage properties of cowpea‐wheat flour blends in bread making. Journal of Food Science, 42, 828–833. https://doi.org/10.1111/j.1365-2621.1977.tb12614.x

[fsn3556-bib-0042] Osundahunsi, O. F. , & Aworh, O. C. (2002). A preliminary study on the use of ‘tempe’ based formula as a weaning diet in Nigeria. Plant Foods for Human Nutrition., 57, 365–376. https://doi.org/10.1023/A:1021805117084 12602942 10.1023/a:1021805117084

[fsn3556-bib-0043] Ragaee, S. , & Abdel‐Aal, E. M. (2006). Pasting properties of starch and protein in selected cereals and quality of their food products. Food Chemistry, 95, 9–18. https://doi.org/10.1016/j.foodchem.2004.p12.012

[fsn3556-bib-0044] Sandhu, K. S. , & Singh, N. (2007). Some properties of corn starches II: Physicochemical, gelatinization, retrogradation, pasting and gel textural properties. Food Chemistry, 101(4), 1499–1507. https://doi.org/10.1016/j.foodchem.2006.01.060

[fsn3556-bib-0501] Savage, G. P. , & Keenan, J. I. (1994). The composition and nutritive value of groundnut kernels In The Groundnut Crop (pp. 173–213). Netherlands: Springer.

[fsn3556-bib-0045] Shazia, Q. , Mohammad, Z. H. , Rahman, T. , & Shekhar, H. U. (2012). Correlation of oxidative stress with serum trace element levels and antioxidant enzyme status in beta thalassemia major patients: A review of the literature. Anemia, 27(7), 923–927. https://doi.org/10.1155/2012/270923 10.1155/2012/270923 PMC3357501 22645668

[fsn3556-bib-0046] Treche, S. , & Mbome, I. L. (1999). Viscosity, energy density and osmolality of gruels for infants prepared from locally produced commercial flours in some developing countries. International Journal of Food Science and Nutrition, 50(2), 117–125. https://doi.org/10.1080/096374899101319 10.1080/096374899101319 10616652

[fsn3556-bib-0047] Udensi, E. A. , Odom, T. C. , Nwaorgu, O. J. , Emecheta, R. O. , & Ihemanma, C. A. (2012). Production and evaluation of the nutritional quality of weaning food formulation from roasted millet and *Mucuna cochinchinesis* . Sky Journal of Food Science, 1(1), 1–5.

[fsn3556-bib-0048] Ukpabi, U. J. , & Ndimele, C. (1990). Evaluation of the quality of Garri produced in Imo State. Nigerian Food Journal, 8, 105–110.

[fsn3556-bib-0502] Verbalis, J. G. , Barsony, J. , Sugimura, Y. , Tian, Y. , Adams, D. J. , Carter, E. A. , & Resnick, H. E. (2010). Hyponatremia‐induced osteoporosis. Journal of Bone and Mineral Research, 25(3), 554–563. 19751154 10.1359/jbmr.090827 PMC3153395

[fsn3556-bib-0049] Voskaki, I. , Arvanitidou, V. , Athanasopoulou, H. , Tzagkaraki, A. , Tripsianis, G. , & Giannoulia‐Karantana, A. (2010). Serum copper and zinc levels in healthy Greek children and their parents. Biological Trace Element Research, 134(2), 136–145. https://doi.org/10.1007/s12011-009-8462-2 19727576 10.1007/s12011-009-8462-2

[fsn3556-bib-0050] Wang, J. C. , & Kinsella, J. E. (1976). Functional properties of novel proteins: alfalfa leaf protein. Journal of Food Science, 41(2), 286–289. https://doi.org/10.1111/j.1365-2621.1976.tb00602.x

[fsn3556-bib-0051] Wheeler, E. L. , & Ferrel, R. E. (1971). A Method for Phytic Acid Determination in Wheat and Wheat Fractions. Cereal Chemistry, 48, 312–320.

[fsn3556-bib-0052] WHO (2004). Guiding principles for feeding infants and young children during emergencies. Geneva: World Health Organization.

[fsn3556-bib-0053] WHO (2012). Guideline: Potassium intake for adults and children. Geneva: World Health Organization. 23617019

[fsn3556-bib-0054] WHO (2016). Guideline: Daily iron supplementation in infants and children. Geneva: World Health Organization. 27195348

[fsn3556-bib-0055] WHO/FAO (2003). Diet, Nutrition and the Prevention of Chronic Diseases. Report of a Joint Expert Consultation. WHO Technical Report Series No. 916. Geneva: World Health Organization. 12768890

[fsn3556-bib-0056] Xu, J. , Zhang, H. , Guo, X. , & Qian, H. (2012). The impact of germination on the characteristics of brown rice flour and starch. Journal of the Science of Food and Agriculture, 92, 380–387. https://doi.org/10.1002/jsfa.4588 21968914 10.1002/jsfa.4588

[fsn3556-bib-0057] Young, D. B. (2001). Role of potassium in preventive cardiovascular medicine. Boston: Kluwer Academic Publishers https://doi.org/10.1007/978-1-4615-1443-5

